# Unlocking the potential of pyroptosis in tumor immunotherapy: a new horizon in cancer treatment

**DOI:** 10.3389/fimmu.2024.1381778

**Published:** 2024-06-14

**Authors:** Qinan Yin, Si-Yuan Song, Yuan Bian, Yiping Wang, Anchen Deng, Jianzhen Lv, Yi Wang

**Affiliations:** ^1^ Department of Pharmacy, Sichuan Academy of Medical Sciences and Sichuan Provincial People’s Hospital, School of Medicine, University of Electronic Science and Technology of China, Chengdu, China; ^2^ Personalized Drug Therapy Key Laboratory of Sichuan Province, School of Medicine, University of Electronic Science and Technology of China, Chengdu, China; ^3^ Baylor College of Medicine, Houston, TX, United States; ^4^ Department of Critical Care Medicine, Sichuan Academy of Medical Sciences and Sichuan Provincial People’s Hospital, School of Medicine, University of Electronic Science and Technology of China, Chengdu, China; ^5^ Department of Neuroscience, Chengdu Shishi School, Chengdu, China; ^6^ School of Pharmacy, Guangxi University of Chinese Medicine, Nanning, China; ^7^ Clinical Immunology Translational Medicine Key Laboratory of Sichuan Province, Center of Organ Transplantation, Sichuan Academy of Medical Science and Sichuan Provincial People’s Hospital, Chengdu, Sichuan, China

**Keywords:** pyroptosis, cancer, gasdermin, immunotherapy, mechanism, Chinese medicinal herbs

## Abstract

**Background:**

The interaction between pyroptosis—a form of programmed cell death—and tumor immunity represents a burgeoning field of interest. Pyroptosis exhibits a dual role in cancer: it can both promote tumor development and counteract it by activating immune responses that inhibit tumor evasion and encourage cell death. Current tumor immunotherapy strategies, notably CAR-T cell therapy and immune checkpoint inhibitors (ICIs), alongside the potential of certain traditional Chinese medicinal compounds, highlight the intricate relationship between pyroptosis and cancer immunity. As research delves deeper into pyroptosis mechanisms within tumor therapy, its application in enhancing tumor immune responses emerges as a novel research avenue.

**Purpose:**

This review aims to elucidate the mechanisms underlying pyroptosis, its impact on tumor biology, and the advancements in tumor immunotherapy research.

**Methods:**

A comprehensive literature review was conducted across PubMed, Embase, CNKI, and Wanfang Database from the inception of the study until August 22, 2023. The search employed keywords such as “pyroptosis”, “cancer”, “tumor”, “mechanism”, “immunity”, “gasdermin”, “ICB”, “CAR-T”, “PD-1”, “PD-L1”, “herbal medicine”, “botanical medicine”, “Chinese medicine”, “traditional Chinese medicine”, “immunotherapy”, linked by AND/OR, to capture the latest findings in pyroptosis and tumor immunotherapy.

**Results:**

Pyroptosis is governed by a complex mechanism, with the Gasdermin family playing a pivotal role. While promising for tumor immunotherapy application, research into pyroptosis’s effect on tumor immunity is still evolving. Notably, certain traditional Chinese medicine ingredients have been identified as potential pyroptosis inducers, meriting further exploration.

**Conclusion:**

This review consolidates current knowledge on pyroptosis’s role in tumor immunotherapy. It reveals pyroptosis as a beneficial factor in the immunotherapeutic landscape, suggesting that leveraging pyroptosis for developing novel cancer treatment strategies, including those involving traditional Chinese medicine, represents a forward-looking approach in oncology.

## 1 Introduction

Cancer has emerged as a significant public health concern and has become one of the primary causes of mortality on a global scale (1). Effective treatments for cancer continue to be a challenge, with limited options available to patients for many years, such as surgery, radiation therapy, and chemotherapy, either as standalone treatments or in combination. Recent advancements in oncology have emphasized targeting tumor pathways and characteristics, including the development of immune-mediated therapies, drugs, and biomolecules (2, 3).

Cancer immunotherapy (CIT) has evolved as a pivotal strategy in oncology, harnessing the immune system to mount a potent response against cancer cells. This approach can provoke immunogenic cell death, fostering durable anticancer immunity through various cell death pathways, including apoptosis, necroptosis, pyroptosis, and ferroptosis (4). The transformative potential of cancer immunotherapy in treating malignancies is frequently limited by low immune response rates and immune-related adverse events. Pyroptosis, an inflammatory form of programmed cell death, has the capability to provoke a robust acute inflammatory response and amplify anti-tumor immunity.

Pyroptosis is a novel form of cell death closely associated with inflammation and innate immunity, characterized by distinct morphological changes such as cell swelling, membrane blistering, DNA fragmentation, and eventual cell lysis (5). This form of cell death has garnered significant attention for its capacity to activate the immune system. Initially observed in macrophages, pyroptosis promotes an anti-inflammatory response by releasing immunogenic cellular components, including injury-associated molecular patterns and cytokines, to combat disease (6). In 2001, as research evolved, it became evident that pyroptosis is fundamentally different from traditional apoptosis. It was at this time that the term “cell pyroptosis” was introduced to describe a form of pro-inflammatory programmed cell death initiated by caspase-1 activation in response to specific pathogens, leading to the disruption of cell membrane integrity and the activation of interleukin 18/1β (IL-18/1β) (7). Further research has identified four main pathways that govern pyroptosis. This process has the potential to transform “cold” tumors, which typically exhibit little or no immune response, into “hot” tumors that are more likely to be recognized and targeted by the immune system (8). Notably, immune cell pyroptosis (ICP) and cancer cell pyroptosis (CCP) occur concurrently in tumor patients, influencing tumor progression (9). The dual impact of pyroptosis activation on cancer genesis is significant. While it can promote tumor growth by modifying the immune microenvironment and evading immune detection, it also has the potential to activate the immune system and enhance CIT efficacy through cytokine release (10). Leveraging pyroptosis’s biphasic effects offers promising avenues for understanding tumor biology and developing novel pyroptosis-based therapeutics.

Furthermore, the application of innovative delivery systems such as nanogels, polymer prodrugs, mesoporous materials, and nanovesicles has broadened the scope for inducing pyroptosis in cancer therapy (8, 11). Recent studies have emphasized the role of Zirconium nanoparticles (ZrNPs) as pyrogenic stimulants in tumor immunotherapy. These nanoparticles significantly increase reactive oxygen species (ROS) levels, activate caspase-1, cleave gasdermin D (GSDMD), and mature interleukin-1β (IL-1β), ultimately leading to cell lysis. *In vivo* experiments have demonstrated that pyroptosis induced by ZrNPs enhances anti-tumor immunity by promoting the maturation and proliferation of dendritic cells (DCs) and T cells, significantly reducing tumor growth and lung metastasis (12). Additionally, researchers are exploring the integration of chemotherapy with photodynamic therapy (PDT) as a novel treatment modality. A new supramolecular nanomedicine has been developed to optimize cancer photochemical chemotherapy by facilitating *in situ* drug release at the tumor site during PDT. This strategy aims to enhance the effectiveness of chemotherapy by fostering synergistic photochemotherapy, which leads to a more potent anti-tumor response while reducing systemic toxicity (13). Moreover, a self-reinforcing supramolecular nanomedical drug (SND) that combines synergistic chemotherapy, photodynamic therapy (PDT), and photothermal therapy (PTT) induces immunogenic cell death (ICD) in tumor cells *in vivo*. This approach markedly suppresses the growth of primary and metastatic tumors and shows an anti-metastatic effect on the liver, all without significant systemic toxicity (14). This article will explore pyroptosis, its mechanisms in tumor immunity, its applications in tumor immunotherapy, and potential roles of traditional Chinese medicine.

## 2 Mechanism of pyroptosis

To better elucidate the role of pyroptosis in tumor immunity, we begin by detailing the primary mechanism of this cell death process. Caspases are pivotal in regulating pyroptosis, a type of programmed necrosis (15). For instance, mouse caspase-1 and caspase-11, as well as human caspase-4 and caspase-5, are instrumental in generating mature IL-1β through the cleavage of IL-1β precursor and subsequent activation of downstream caspase-3 (16, 17). The execution of pyroptosis depends not only on the activation of inflammatory caspases but also involves the Gasdermin (GSDM) protein family (18). Humans have six GSDM variants: GSDMA, GSDMB, GSDMC, GSDMD, GSDME, and PJVK (also known as DFNB59 or GSDMF), with GSDMD recognized in 2015 as a key factor in inflammasome-induced pyroptosis. Apart from PJVK, other GSDM family proteins are distinguished by three structural domains: the GSDM N-terminal (GSDM-NT) domain, a linker region, and the GSDM C-terminal (GSDM-CT) domain (19). It has been established that PJVK does not participate in pore formation or pyroptosis (20). Activation by caspases results in the cleavage of GSDMD, releasing gasdermin-N and -C domains, where the gasdermin-N domain binds to and perforates the cell membrane, causing cell swelling and lysis (21, 22).

**Figure 1:**
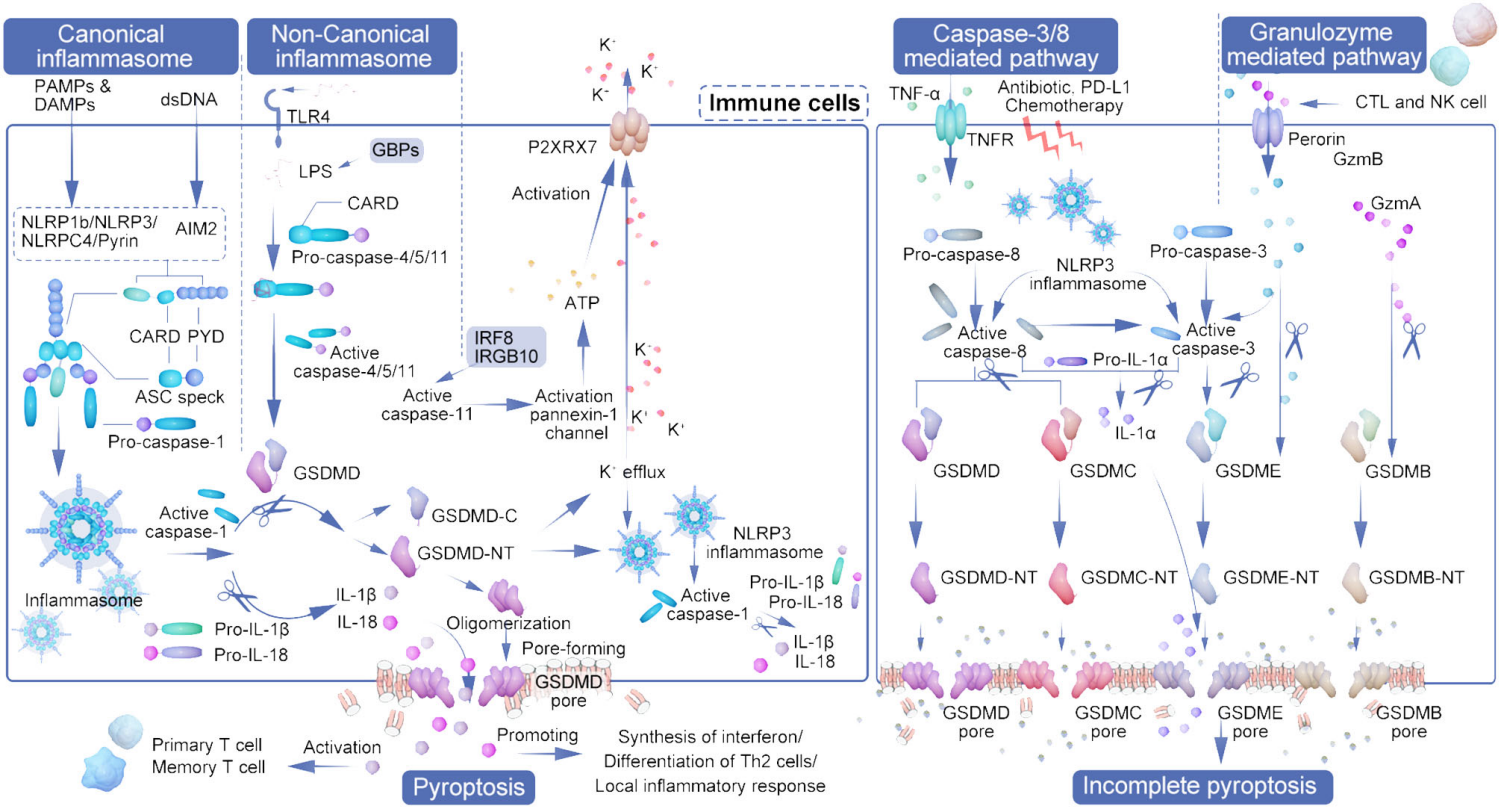
The interplay of pyroptosis pathways and immune cell activation in tumor immunity. The diagram illustrates the orchestrated pathways of pyroptosis, a form of cell death that contributes to tumor immunity by engaging various immune cells and inflammatory processes. Initiation of pyroptosis can occur through different mechanisms: the canonical inflammasome pathway, which is activated by DAMPs and PAMPs leading to NLRP3 inflammasome assembly and caspase-1 activation; and the non-canonical inflammasome pathway, initiated by factors such as LPS and recognized by TLR4, activating caspase-4/5/11. These pathways converge on the cleavage of GSDMD, resulting in the formation of pores in the cell membrane and cell lysis. CTLs and NK cells secrete granzymes that cleave GSDME and GSDMB, further propagating pyroptosis. Active caspase-3, influenced by signals such as TNF-α, antibiotics, and chemotherapy, also contributes to the activation of GSDME and GSDMC, leading to cell rupture. The outcome is the release of pro-inflammatory cytokines (IL-1β, IL-18, IL-22, HMGB1) and danger signals that prompt the recruitment and activation of various immune cells, including dendritic cells (DCs), CD4+ T cells, CD8+ T cells, and myeloid-derived suppressor cells (MDSCs). This immunological response culminates in a heightened anti-tumor activity, as the activated immune cells induce further pyroptosis in the cancer cells, creating a feedback loop that amplifies the anti-tumor immune response. The representation captures the complexity of pyroptosis and its significant role in enhancing the efficacy of immune-mediated cancer therapies.

Among the gasdermins, GSDMD and GSDME are the most thoroughly studied in terms of their role in pyroptosis induction (23). The pathway mediated by GSDMD encompasses both canonical and non-canonical inflammasome pathways. This review will delineate the four recognized pyroptosis induction pathways, as illustrated in **Figure 1**, dividing the pyroptosis process into four principal phases: signal detection, signal transmission, activation of the executioner, and the execution of pyroptosis (24).

### 2.1 Canonical pathway

The canonical inflammasome pathway, predominantly active in immune cells, serves as a defense mechanism against pathogens, utilizing caspase-1 activation in response to pathogen-associated molecular patterns (PAMPs) and damage-associated molecular patterns (DAMPs) such as ATP and cholesterol (25). Inflammasomes, critical for innate immunity, inflammation, and cell death, play significant roles in cancer development and management. The initiation and regulation of the innate immune response are orchestrated by a variety of encoded pattern recognition receptors (PRRs), such as Toll-like receptors (TLRs), RIG-I receptors (RLRs), NOD-like receptors (NLRs), AIM2-like receptors (ALRs), C-type lectin receptors (CLRs), and other DNA sensors. Activation of these PRRs leads to the activation of NF-KB, type I interferon (IFN), and other inflammasome signaling pathways, resulting in the production of various pro-inflammatory and antiviral cytokines and chemokines that subsequently stimulate an adaptive immune response (26). Additionally, NLRs (NLRP1b, NLRC4, NLRP3) and AIM2, which are equipped with caspase recruitment cytoplasmic dsDNA, crystals, and toxins. Specifically, pyrin targets host GTPases that are affected by bacterial toxins (27, 28). Caspase-1, a critical cysteine protease in innate immunity, is activated by various signals, including pathogens, stress, and injury, through inflammasome oligomerization. Its activation not only facilitates the release of pro-inflammatory cytokines but also mediates pyroptosis, driving an immune response for pathogen eradication (29). The inflammasome, comprised of NLR and adaptor protein ASC, facilitates interaction with procaspase-1 via the caspase recruitment domain, leading to self-cleavage and activation of caspase-1. Caspase-1 plays a crucial role in the maturation of proinflammatory cytokines IL-1β and IL-18 and the initiation of pyroptosis (30). Moreover, it is important to note that IL-1β and IL-18 can also be cleaved by caspase-1 into mature forms, which are then secreted through the pores formed by GSDMD-NT (31).

### 2.2 Non-canonical inflammasome pathway

The non-classical inflammasome pathway is distinguished by its independence from caspase-1, caspase-3, or caspase-8. Instead, it is directly activated by caspase-11 in mice and caspase-4/5 in humans when interacting with lipopolysaccharide (LPS) from the cell walls of Gram-negative bacteria (29, 32). This interaction facilitates the activation of these caspases through direct engagement with their CARD domains, bypassing the conventional activation mechanisms (27, 28, 33). Furthermore, the High mobility group box-1 protein (HMGB1), by binding to LPS, facilitates its internalization into macrophages and endothelial cells through the advanced glycation end-products (AGER) receptor, leading to cytoplasmic LPS release and subsequent caspase-11 activation due to HMGB1’s interaction with the phospholipid bilayer (34). The NLRP3 inflammasome acts downstream in this pathway, specifically facilitating the maturation of IL-1β following cytoplasmic LPS detection by caspase-4/5 (35). Intracellular LPS directly interacts with caspase-4/5/11, leading to the cleavage of GSDMD and the subsequent release of its N-terminal and C-terminal fragments (36–39). Toll-like receptor 4 (TLR4) acts as the membrane-bound receptor that initially detects LPS. Additionally, guanylate binding proteins (GBPs), which are induced by interferon, play a critical role in the cytoplasmic regulation of this unconventional inflammasome activation pathway and subsequent pyroptosis (40).

While caspase-4/5/11 themselves do not directly mature IL-1β and IL-18, caspase-11’s activation of the pannexin-1 channel facilitates ATP release. This, in turn, activates the P2X purine receptor 7 (P2RX7), leading to K+ efflux and NLRP3 inflammasome activation, thereby indirectly initiating caspase-1 activation similar to the classical pathway (41, 42). In certain cell subsets, IL-1β and IL-18 secretion is thus indirectly induced in an NLRP3-dependent manner (43). Intriguingly, interferon and NF-κB signaling pathways, including factors such as IRF8 and IRGB10, also influence caspase-11 activation (44). The non-canonical pathway, particularly through caspase-4/11 activation by cytoplasmic LPS or Gram-negative bacteria (45), prompts neutrophil pyroptosis in a GSDMD-dependent fashion. This process is closely linked to the extrusion of neutrophil extracellular traps (NETs), associating non-canonical inflammasome-induced pyroptosis with NETosis—a specialized form of neutrophil death in response to pathogen invasion (46).

### 2.3 Caspase-3/8-mediated pathway

Distinct from the pathways previously discussed, the effector in pyroptosis mediated by caspase-3/8 is GSDME, a protein sharing the gasdermin-N domain characteristic of the GSDM family (23). Caspase-3 resides as an inactive proenzyme within the cytoplasm and catalyzes the cleavage of peptide bonds following aspartate residues at C-terminal cysteine sites. Upon activation, caspase-3 induces GSDME, which is located downstream, to produce an N-terminal domain that creates openings in the cellular membrane, leading to cellular swelling and rupture. This process releases inflammatory mediators and DAMP molecules. Increased GSDME expression following chemotherapy enhances the activation of caspase-3, which then contributes to the pyroptotic pathway by cleaving GSDME. This cleavage releases its N-terminal fragment, perforating the cell membrane and initiating cell death (43, 47, 48).

Caspase-8, noted for its versatility, is integral to various cellular processes including apoptosis, inflammation, and necrosis. It can initiate apoptosis by cleaving caspase-3, induce pyroptosis through GSDMD cleavage, and regulate necrosis by targeting RIPK1 and RIPK3 (49, 50). Strategically positioned within the inflammasome activation pathways, caspase-8 exerts substantial influence both upstream and downstream, directly or indirectly triggering inflammasome activation. In situations where caspase-1 or GSDMD activities are inhibited, caspase-8 activation can ensue, potentially leading to an alternative inflammatory cell death pathway through the recruitment of the inflammasome complex (51). This mechanism enables modulation of inflammation at lower levels or in response to pathogenic attempts to inhibit inflammatory processes (52, 53).

Moreover, caspase-8 facilitates pyroptosis by cleaving GSDMC and GSDME. It’s revealed that caspase-8 can trigger pyroptosis in breast cancer cells through the caspase-8/GSDMC pathway upon stimulation by TNF-α or chemotherapy agents, highlighting its role in cancer cell pyroptosis (54). A novel pathway involves α-ketoglutaric acid (α-KG) promoting pyroptosis via caspase-8-mediated GSDMC cleavage, where a cell-permeable α-KG derivative enhances ROS production, leading to plasma membrane oxidation and subsequent pyroptotic cell death (55).

In macrophages lacking caspase-1/11, NLRP3 inflammasome can still activate caspase-3/8 to cleave GSDME, inducing a unique form of pyroptosis. This variant is notable for not releasing IL-1β but rather IL-1α, illustrating an alternative pathway for pyroptotic signaling (56).

### 2.4 Granzyme mediated pathway

Granzymes, serine proteases released by cytotoxic T lymphocytes (CTLs) and natural killer (NK) cells, these enzymes play a crucial role in initiating pyroptosis by selectively targeting substrates within the targeted cells (57). Groundbreaking research by Shao Feng’s team in 2020 unveiled that Granzyme A (GZMA), emitted by these lymphocytes, triggers cell death by cleaving GSDMB at specific sites (Lys229/Lys244), releasing GSDMB-N fragments that perforate the cell membrane, thus initiating pyroptosis (58, 59). It was observed that only the N-terminal fragments of GSDMB subtypes 3 and 4 induce pyroptosis, whereas subtypes 1, 2, and 5 do not, with non-cytotoxic GSDMB-NTs inhibiting the action of cytotoxic ones in a dominant negative fashion (60).

Simultaneously, research by Judy Lieberman’s team identified that granzyme B cleaves GSDME directly after D270, highlighting GSDME’s role beyond its initial association with hereditary deafness. GSDME, frequently downregulated in cancer compared to normal tissues, acts as a tumor suppressor and is crucial in caspase-3 and granzyme B-mediated cell death, implicating its significance in innate immunity, tissue damage, cancer, and hearing loss. This positions GSDME as a potential biomarker for cancer diagnosis, monitoring, and evaluating response to chemotherapy and immunotherapy, capable of inciting caspase-independent pyroptosis in GSDME-positive cells (61, 62). Granzyme B has the dual capability of directly cleaving the GSDME D270 site, thereby shifting the immune cell-mediated cell killing mechanism to pyroptosis, and of indirectly activating caspase-3 (23). Moreover, GSDME can be specifically cleaved and activated by caspase-3, ultimately leading to pyroptosis. Additionally, Granzyme B from cytotoxic lymphocytes can induce pyroptosis through caspase-independent cleavage of GSDME. Similarly, Granzyme A from natural killer cells and cytotoxic T lymphocytes is capable of cleaving GSDMB, which also results in pyroptosis (63).

These discoveries redefine pyroptosis, challenging the prior notion that its activation is solely caspase-dependent. Furthermore, CAR-T cells have been shown to prompt rapid caspase-3 activation in target cells through granzyme B release, cleaving GSDME and inducing extensive pyroptosis in susceptible cells, underscoring the potential of GSDME and caspase-3 as targets for cancer therapy (64, 65).

## 3 Role of pyroptosis in tumor immunity

Pyroptosis, an inflammatory form of programmed cell death, plays a crucial role in disrupting the tumor microenvironment’s immunosuppressive state and enhancing anti-tumor immune responses (66). This process involves tumor cells drawing in immune cells through the release of danger signals during pyroptosis, thereby facilitating the immune-mediated destruction of tumor cells. The release of cancer antigens and inflammatory mediators through pyroptosis aids in antigen presentation by dendritic cells, leading to the activation of T cells. This process entails the activation of NK cells and cytotoxic T lymphocytes, which secrete granzymes A and B to target GSDMB and GSDME in tumor cells, ultimately inducing pyroptosis and bolstering anti-tumor immunity. This creates a feedback loop that serves to amplify both processes (67, 68). Research has shown that even a minor percentage of tumor cells undergoing pyroptosis can significantly enhance tumor clearance, underscoring the powerful role of anti-cancer immunity in this context (69).

Pyroptosis enhances T cell infiltration within tumors and boosts T cell efficacy. The role of NLRP3 inflammasomes in modulating the tumor microenvironment is significant, particularly in how they influence the recruitment of myeloid-derived suppressor cells (MDSCs) and the suppression of anti-tumor T cell activity following dendritic cell vaccination. NLRP3 expression in tumor-associated macrophages is linked to the promotion of an immunosuppressive milieu through the polarization of CD4+ T cells, driven by IL-1β (57, 70). Research findings reveal that tumors experiencing cancer cell pyroptosis (CCP) demonstrate significant infiltration by CD4+ and CD8+ T cells, resulting in immune-dependent tumor regression (69).

In tumors expressing GSDME, an increase in the quantity and functionality of dendritic cells, T cells, and NK cells has been observed, underscoring the importance of GSDME in immune-mediated tumor suppression—a phenomenon absent in immunodeficient models. Dendritic cells are central to anti-tumor immunity, driving long-term protective T cell responses and facilitating effective anti-tumor immunity, including against tumors resistant to PD-1 inhibition, through the cDC1 subpopulation and IL-1ß-dependent mechanisms (71).

GSDME’s silencing or mutation in various cancers highlights its significant tumor-suppressive role. The production of IL-22, dependent on NLRP3 inflammasome activation, and the role of HMGB1 as an inflammatory stimulant, underline complex interactions within the tumor microenvironment that foster anti-tumor responses. HMGB1’s interaction with TLR4, for instance, triggers macrophage activation and innate immune responses, contributing to anti-tumor efficacy (72).

**Figure 2:**
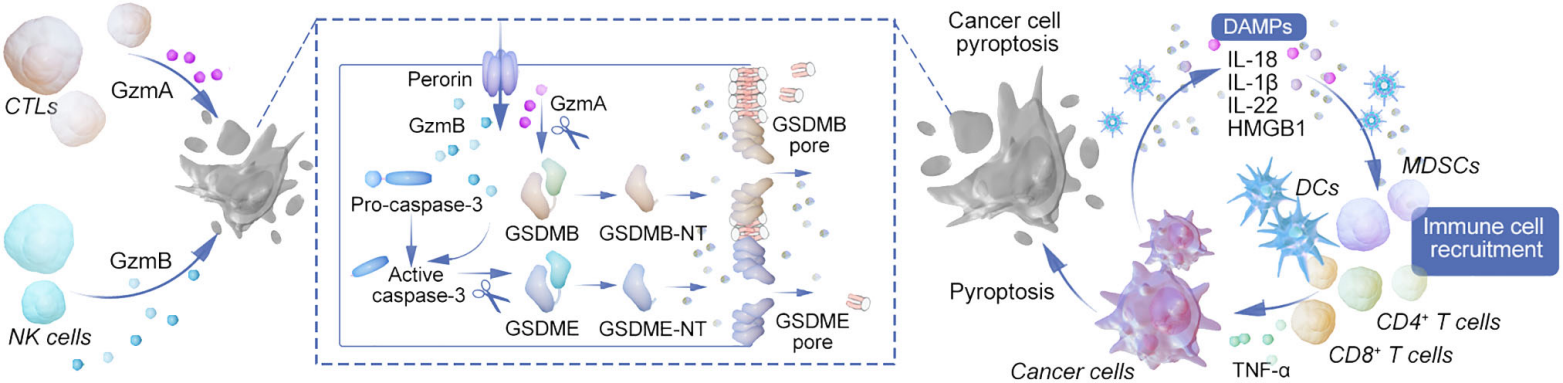
Mechanisms of pyroptosis in modulating tumor immunity and immunotherapy. This diagram provides a comprehensive overview of the various pathways leading to pyroptosis and their intricate connections with immune cell functions and tumor immunotherapy. The canonical inflammasome pathway is initiated by pathogen-associated molecular patterns (PAMPs) and damage-associated molecular patterns (DAMPs), leading to the assembly of the inflammasome complex and activation of caspase-1. Subsequently, caspase-1 facilitates the maturation and secretion of IL-1β and IL-18, promoting inflammation. The non-canonical inflammasome pathway, triggered by LPS recognition through TLR4 and GBPs, activates caspase-4/5/11, which directly cleaves GSDMD, contributing to the lytic form of cell death known as pyroptosis. Caspase-3/8 mediated pathway illustrates the role of external triggers such as TNF-α, antibiotics, and chemotherapy in activating caspase-8, which then cleaves GSDMD or GSDMC, leading to pyroptosis and the release of IL-1α. Granzyme-mediated pathway depicts how cytotoxic cells, such as CTLs and NK cells, release granzymes that cut GSDME and GSDMB, inducing pyroptosis in target cells. The figure also highlights the release of immune cell attractants and the promotion of adaptive immune responses through antigen presentation, leading to the recruitment of primary and memory T cells. This process creates a feedback loop, enhancing the anti-tumor immune response and potentially synergizing with immunotherapeutic strategies such as CAR-T and checkpoint blockade therapies. The interconnected pathways underscore the complex role of pyroptosis in shaping the tumor microenvironment, influencing tumor regression, and informing the development of novel cancer treatment strategies.

Conversely, IL-1β and IL-18 may facilitate tumor cell evasion from immune responses, highlighting the complex roles these cytokines play in the tumor microenvironment (10). Additionally, tumor cells undergoing pyroptosis can induce similar cell death in immune cells such as myeloid-derived suppressor cells (MDSCs), or trigger hyperactivation in tumor-associated macrophages (TAMs) and dendritic cells (DCs), thereby shaping the immune landscape within tumors (73). See **Figure 2**.

Furthermore, Yang, Bowen et al. introduced the term “Reactive Oxygen Science,” a growing field focused on the chemical mechanisms, biological impacts, and potential nanotherapeutic applications of reactive oxygen species. The crucial role of reactive oxygen species in facilitating the immunogenic pyroptosis of tumor cells has been emphasized (74). In the realm of photodynamic therapy (PDT), a novel and mainstream reactive oxygen species-based treatment, the use of small molecule photosensitizers involving cationic units or ligands derived from cell membrane surface receptors can modify the anchoring capabilities of the cell membrane, altering the mode of cell death towards pyroptosis. For nano photosensitizers, inducing photodynamic therapy at different stages of endocytosis in tumor cells with high gasdermin E (GSDME) expression leads to diverse cell death patterns. Notably, initiating PDT in early endosomes has been shown to provoke GSDME-mediated pyroptosis through the activation of phospholipase C (PLC) signaling (75).

## 4 Application of pyroptosis in tumor immunotherapy

In clinical oncology, the primary modalities for combating tumors include surgical intervention, chemotherapy, radiotherapy, immune checkpoint inhibitors, and targeted therapies. Additionally, some cancer treatment protocols enhance anti-tumor immune responses by incorporating strategies such as pyroptosis and modulation of the tumor immune microenvironment. Tumor immunotherapy capitalizes on the body’s immune system to target and destroy cancer cells, either by amplifying natural anti-tumor responses or by utilizing engineered immune cells. Among the available treatments, immune checkpoint blockade (ICB) and chimeric antigen receptor T-cell (CAR-T) therapy have shown significant efficacy in managing various cancers, including lung cancer, melanoma, and B-cell lymphoma (76). Pyroptosis plays a crucial role in these therapies, influencing immunogenicity either by suppression or enhancement, thereby leading to substantial progress in cancer treatment. This process is distinguished by its unique ability to disrupt cell membranes, which triggers the release of inflammatory mediators and amplifies systemic immune responses (77).

A novel approach involves administering GSDMA3-NT into tumor cells alongside PH-BF3, a cancer imaging agent that targets tumor cells for selective desilication and cleavage of silyl ether-containing connectors. This method ensures the delivery and release of GSDMA3-NT within tumor cells, inducing pyroptosis in about 10% of the cell population, sufficient to reject the entire 4T1 breast tumor graft. However, this effect is absent in immunodeficient or T-cell-depleted mice, underscoring the importance of pyroptosis in activating anti-tumor immunity (69).

Pyroptosis is instrumental in cancer therapy, with one study revealing an increased expression of 17 pyroptosis-related genes in highly immunogenic tumors, whereas tumors with low immune activity showed reduced pyroptosis. Furthermore, across 30 types of cancer, pyroptosis was positively correlated with immune infiltration and immune-related characteristics, indicating its significant association with tumor immunity and prognosis. This positions pyroptosis as a promising target for immunotherapy (78).

### 4.1 CAR-T

Chimeric antigen receptor T-cell (CAR-T) therapy represents a groundbreaking advancement in precision cellular immunotherapy, designed to target tumor-specific neoantigens for personalized cancer treatment. By harnessing the natural anti-tumor properties of T cells, this technology equips T cells to specifically recognize and eliminate tumor cells through neoantigen recognition. The effectiveness of these engineered CAR T cells has shown significant promise, particularly in treating hematological malignancies (79). These cells can induce pyroptosis in tumor cells by activating the caspase-3 and GSDME pathways following the release of perforin and granzyme B. This pyroptotic process may also activate caspase-1 and lead to the fragmentation of GSDMD within macrophages, resulting in cytokine secretion and potentially triggering cytokine release syndrome (CRS), a severe adverse event marked by fever, hypotension, and respiratory distress (64, 80). Studies suggest that cytokine release syndrome (CRS) can be managed with IL-1 blocking therapies, such as anakinra, although it is crucial to carefully time these treatments to avoid interfering with the cytokine release necessary for effective immune activation (81).

Innovatively, CAR T cells have been demonstrated to cause GSDME-dependent pyroptosis specifically in leukemia cells through the action of granzyme B, a mechanism distinct from that observed with native CD8+ T cells. This specificity suggests that using CAR T cells in conjunction with immune checkpoint inhibitors (ICIs) and pyroptosis inducers might offer a targeted approach to solid tumor treatment without the severe side effects typically associated with broad-spectrum immune activation (57, 82). In experiments, 293 T cells engineered to express CD19 and GSDMB, when exposed to human anti-CD19 CAR T cells, underwent GSDMB cleavage leading to pyroptosis, thus enhancing anti-tumor immunity (83).

Further advancements include the development of a novel chimeric co-stimulatory conversion receptor (CCCR) in CAR-T NK cells. This receptor integrates the extracellular domain of PD-1 with the transmembrane and intracellular domains of NKG2D and 41BB, respectively. Designed to transform the inhibitory PD-1/PD-L1 interaction into a stimulatory signal, this innovative receptor aims to boost the immunosuppressive efficiency of PD-1 targeted therapies (84), offering a new avenue for enhancing CAR-T cell efficacy against cancer.

### 4.2 Immune checkpoint inhibitor

Immune checkpoint inhibitor (ICI) is a highly sought-after immunotherapy in the treatment of tumors due to its demonstrated efficacy. Nevertheless, challenges persist in ICI treatment, such as low response rates and a deficiency in reliable predictors of treatment effectiveness (85–87). Emerging research highlights a synergistic relationship between cell pyroptosis and ICI, with findings that pyroptosis induction may render ICI-resistant tumors susceptible to treatment. This synergy stems from the disruptive effects of pyroptosis on the tumor microenvironment, facilitating an influx of lymphocytes that perpetuate a cycle of immune-mediated tumor cell destruction. ICIs, by targeting tumor neoantigens, bolster the anti-tumor immune response, a critical factor in overcoming immunosuppressive barriers within tumor development (69).

Studies have demonstrated a positive correlation between pyroptosis and immune infiltration across various cancers, suggesting that pyroptosis directly influences immune checkpoint molecule expression, aligning with therapeutic strategies aimed at converting immunologically “cold” tumors into “hot” ones. This relationship underscores the potential of pyroptosis as a biomarker for immunotherapy response and supports the integration of pyroptosis inducers with immunotherapeutic agents for enhanced cancer treatment efficacy (88).

Research also indicates that tumor-associated macrophages (TAMs) and the chemokines they produce play a pivotal role in recruiting CD8+ T and NK cells following inflammasome activation and pyroptosis, further establishing the connection between pyroptosis and immune checkpoint blockade efficacy (89). The expression of GSDMB within tumors has been shown to significantly enhance the effectiveness of ICB therapy, highlighting pyroptosis’s role in augmenting anti-tumor immunity (90).

The immune checkpoint protein PD-L1 plays a critical role in suppressing anti-tumor immunity by interacting with PD-1. Targeting this interaction has led to promising results in cancer therapy. Recent research has investigated how pyroptosis can augment anti-PD-1 therapy, revealing that nuclear PD-L1 can induce pyroptosis through its interaction with phosphorylated STAT3. These insights elucidate the mechanisms through which cancer cells fend off pathogen infections and underscore the potential therapeutic applications of pyroptosis in treating cancer (24, 91).

Further, the development of IBI315, a bispecific antibody targeting PD-1 and Her2, has been shown to initiate GSDMB-mediated pyroptosis in tumor cells, thereby activating and recruiting T cells to the tumor site, establishing a feedback loop that enhances tumor cell eradication (92). Additional research in the BRAFV600E/PTEN melanoma model has revealed that NLRP3-mediated recruitment of immunosuppressive cells can foster anti-PD1 resistance, while also promoting pyroptosis and bolstering anti-tumor immunity (93).

This detailed examination of pyroptosis within the framework of cancer immunotherapy, focusing on its interactions with immune checkpoint inhibitors and potential enhancements to treatment strategies through modulation of the tumor immune microenvironment, highlights the complex interplay between mechanisms of cancer cell death and immune responses. This analysis establishes a foundation for novel therapeutic interventions in oncology.

## 5 Potential Chinese medicine

In the realm of potential Chinese medicine, Quercetin—a plant pigment prevalent in tea and onions—serves as a potent antioxidant. The name “Quercetin” originates from the Latin word “quercetum,” meaning oak. It is recognized for its extensive therapeutic properties, including anti-allergic, anti-inflammatory, anti-carcinogenic, cardio-protective, anti-tumor, antiviral, anti-diabetic, immunomodulatory, anti-hypertensive, and gastroprotective effects (94, 95). Recent studies by Liu et al. (96) have illustrated quercetin’s beneficial effects on the tumor microenvironment, inhibiting tumor metastasis and promoting autophagy in cancer cells. Specifically, quercetin has been shown to suppress the proliferation of gastric cancer cells by activating the pyroptosis pathway, as evidenced by increased expression of pyroptosis-related genes (GSDMD, GSDME, Cleaved CASP1, NLRP3) in treated AGS cells. This indicates quercetin’s potential to trigger critical genes involved in pyroptosis, particularly GSDMD, leading to the enhanced clearance of gastric cancer cells. Additionally, quercetin’s ability to upregulate caspase-3 and PARP in AGS cells suggests its role in inducing apoptosis (97). Quercetin effectively inhibits the growth of colon cancer cells by inducing pyroptosis through GSDMD activation. Treatment with quercetin up-regulates the expression of NIMA-related kinase 7 (NEK7), which promotes the assembly of the NLRP3 inflammasome and facilitates the cleavage of GSDMD (98). Furthermore, quercetin significantly reduces neuronal death caused by breast cancer by down-regulating the PYD and card domain containing protein (ASC), NLRP3, and caspase-1, and it reverses the suppression of neuronal activity induced by breast cancer, thereby significantly alleviating symptoms (99).

Triptolide, a diterpenoid compound derived from Tripterygium wilfordii Hook. F., possesses remarkable pharmacological effects. Studies have shown its potent inhibitory impact on various types of solid tumors (100). TPL’s mechanism involves inhibiting mitochondrial HK-II and aerobic glycolysis, promoting GSDME-mediated pyroptosis, and facilitating the translocation of mitochondrial proteins BAX/BAD, thereby targeting head and neck cancer cells. The inhibition of GSDME expression can mitigate TPL’s cytotoxic effects on cancer cells, with TPL treatment reducing c-myc and HK-II expression and activating the BAD/BAX-caspase 3 pathway to induce GSDME lysis (101).

Baicalin, identified as 5,6,7-trihydroxyl flavone, is the principal bioactive constituent present in Scutellaria baicalensis and is accountable for the majority of its advantageous physiological effects. This flavonoid has been demonstrated to regulate a variety of physiological processes, such as antioxidant, antiviral, anti-inflammatory, anti-angiogenic, and anticancer properties (102). Baicalein, a key flavonoid of Scutellaria baicalensis, exhibits promise for the treatment of gastric cancer by activating the NLRP3 inflammasome and triggering pyroptosis in a manner dependent on dosa (103). Meanwhile, research into baicalin’s effects on pancreatitis emphasizes its ability to alleviate hyperlipidemic pancreatitis by inhibiting the NLRP3/caspase-1 pathway (104).

Luteolin, a flavonoid with the chemical formula 3,4,5,7-tetrahydroxy flavone, is found in a variety of vegetables, herbs, and fruits. This compound exhibits anticancer properties and has demonstrated efficacy against a range of human malignancies, including lung, breast, glioblastoma, prostate, colon, and pancreatic cancers (105). Additionally, luteolin has been shown to increase the expression levels of IL-1β, GSDMD, and caspase-1 in colorectal cancer tissues, highlighting its potential therapeutic value (106). Similarly, the primary alkaloid from *Coptis chinensis* influences the NLRP3 inflammasome pathway, decreasing the viability and migration of MDA-MB-231 cells while downregulating key proteins in the NLRP3 cascade (107). Ajmalicine (AJM), an alkaloid extracted from *Coptis yunnanensis* root, has been shown to promote pyroptosis in H22 cells, increasing the pyroptosis rate, up-regulating the expression of TNF-α, IL-1β, and IL-6, enhancing membrane pore openness, and boosting ROS expression. AJM also enhances the expression of caspase-3 and GSDME. In animal studies, AJM has been effective in inhibiting tumor growth by activating the ROS through an atypical caspase-3-GSDME pyroptosis pathway, thus playing an antitumor role (108).

Betulinic acid (BA), a widely distributed pentacyclic triterpene with the Wolf alkane structure, is recognized as a promising natural agent for the prevention, suppression, and management of various human malignancies (109). Research indicates a significant increase in caspase-1 expression and a reduction in ki67 expression in treated models, suggesting that betulinic acid boosts cisplatin’s effectiveness through apoptosis induction (110).

Oridonin, an ent-kaurane diterpenoid derived from Rabdosia rubescens, is a botanical species commonly known as donglingcao in China. It has been historically utilized in traditional East Asian medicine for its therapeutic properties in addressing inflammation and cancer (111). Recent research has demonstrated that oridonin exhibits anti-tumor properties through its regulation of pyroptosis via multiple mechanisms. Oridonin has been shown to suppress pyroptosis by inhibiting caspase-1, a key enzyme involved in the activation of pyroptosis in canonical pathways. Furthermore, oridonin can also inhibit pyroptosis by targeting NLRP3, a crucial inflammasome involved in the activation of pyroptosis in non-canonical pathways. Conversely, oridonin can induce pyroptosis by activating caspase-3 and caspase-8. Additionally, oridonin plays a pivotal role in the regulation of pyroptosis by inhibiting ncRNA and NLRP3 pathways while promoting the accumulation of ROS (112).

This body of research highlights the various mechanisms through which natural compounds influence cancer treatment, ranging from inducing pyroptosis to enhancing immune responses, and offers promising avenues for future cancer therapies. Currently, there is limited research on the inhibition of tumor immunity by traditional Chinese medicine through pyroptosis, and no literature directly compares traditional Chinese medicine with Western medicine in this research area. Nonetheless, existing studies indicate that some botanical drugs, such as ginkgetin and betulinic acid, can enhance the cleavage of caspase-3 and GSDME in cancer cells exposed to paclitaxel, thereby inducing higher levels of pyroptosis and potentially overcoming resistance to cancer chemotherapy (113).

## 6 Summary and prospect

The complex interplay between pyroptosis and tumor immunity underscores a dynamic that both promotes and inhibits tumor progression, reflecting the intricacy of pyroptosis in cancer research. As a focal point for developing innovative immunotherapeutic strategies, pyroptosis, characterized by the activation of the Gasdermin family, is recognized as a key mechanism in cancer cell death, suggesting significant implications for diagnostics and therapeutics. In particular, the involvement of caspase-6 in mediating necroptosis through GSDMD and its role in PANoptosis are of considerable importance in tumorigenesis and antitumor immunity, presenting new opportunities for cancer treatment (114).

Recent advancements have illustrated pyroptosis’s promise in enhancing tumor immunotherapy, including its integration with CAR-T therapy and immune checkpoint blockade, offering innovative approaches to refine immunotherapeutic outcomes. Innovations such as bionic nanoparticles, which synergize indocyanine green and decitabine to trigger pyroptosis, have shown to attenuate immunosuppression and bolster systemic immunity, effectively combating primary and metastatic tumors (115). Additionally, the generation of reactive oxygen species (ROS) during photodynamic therapy (PDT) has been identified as a potent inducer of pyroptosis, with new photosensitizers capable of triggering immunogenic cancer cell pyroptosis, broadening the horizon for PDT applications (116, 117).

Explorations into traditional Chinese medicine have also uncovered components capable of inducing pyroptosis, although research in this area remains sparse, warranting further investigation. Despite the recognized antitumor properties of pyroptosis, many questions persist, particularly regarding the functional roles of gasdermins in tumors and their impact on the tumor immune microenvironment. Addressing challenges such as minimizing collateral tissue damage from pyroptosis, such as intestinal and renal toxicity, is crucial for advancing clinical applications (118).

Beyond oncology, the implications of pyroptosis extend to cardiovascular (119) and endocrine systems (120), among others, illustrating its broad potential in disease treatment and management (121). As research progresses, the expansive application of pyroptosis across various medical fields continues to hold great promise.

## Author contributions

QY: Writing – original draft. S-YS: Writing – original draft. YB: Writing – original draft. YPW: Writing – original draft. AD: Data curation, Writing – original draft. JL: Supervision, Writing – review & editing. YW: Conceptualization, Investigation, Supervision, Writing – review & editing.
